# Entrepreneurial Passion and Entrepreneurial Success—The Role of Psychological Capital and Entrepreneurial Policy Support

**DOI:** 10.3389/fpsyg.2022.792066

**Published:** 2022-02-25

**Authors:** Wei Hu, Yan Xu, Fuqiang Zhao, Yun Chen

**Affiliations:** ^1^School of Management, Wuhan University of Technology, Wuhan, China; ^2^School of Public Administration and Policy, Renmin University of China, Beijing, China

**Keywords:** entrepreneurial passion, psychological capital, entrepreneurial policy support, entrepreneurial success, social information processing theory, resource conservation theory

## Abstract

Entrepreneurship success is the ultimate goal pursued by entrepreneurs, and entrepreneurial passion is also considered an indispensable and important element on the road to entrepreneurial success. However, the internal influence mechanism of entrepreneurial passion on entrepreneurial success is still insufficient in academic circles. In view of this, based on the theory of social information processing, this research analyses the internal mechanism of entrepreneurial passion through individual psychological capital on entrepreneurial success and the promotion of external entrepreneurial policy support. Through a multi-time and multi-source questionnaire survey of 455 entrepreneurs in entrepreneurship parks and entrepreneurship centers in Wuhan, Guangdong, Shanghai, and other places, the research results show that entrepreneurial passion can increase individual psychological capital and positively affect entrepreneurial success; psychological capital mediates the relationship between individual entrepreneurial passion and entrepreneurial success. Entrepreneurship policy support at the environmental level can promote the effect of entrepreneurial passion on the accumulation of psychological capital, and promote the transformation of entrepreneurial passion into entrepreneurial success through psychological capital. The above research results are helpful to the accumulation of entrepreneurs’ psychological capital and provide a useful reference for entrepreneurial success.

## Introduction

Entrepreneurship, as an important path to achieve individual wealth accumulation, increase social employment, and promote social prosperity and development (Reynolds et al., 2004), has always been a research hotspot in the fields of management, organizational behavior, and psychology. As the ultimate goal of entrepreneurs, entrepreneurial success has always been paid attention to by academia. In China, although the entrepreneurial activity index is much higher than other innovation-driven countries, the entrepreneurial success rate has not been high (Yang et al., 2019). How to increase the success rate of entrepreneurship, promote national entrepreneurship support policies, and maintain entrepreneurial passion has become a hot topic for scholars. Therefore, it is particularly necessary to explore the influencing factors of entrepreneurial success.

Although the research on entrepreneurial passion as the antecedent of entrepreneurial success has been extensively tested (Zhang et al., 2019), there are different perspectives. Self-regulation theory points out that when individuals believe that the goal is valuable or the individual enjoys the process of pursuing the goal, they can insist on pursuing the goal (Ma et al., 2017). More specifically, entrepreneurial passion enables entrepreneurs to identify with the activities they participate in, and persist in entrepreneurship more persistently until they succeed. The resource conservation theory states that individual entrepreneurial passion can prompt them to patch up social resources to meet the basic needs of entrepreneurial success (Ma et al., 2017). Although a large number of studies have explored the inner relationship between entrepreneurial passion and entrepreneurial success from different perspectives, they have not involved individual psychological changes, such as entrepreneurial self-confidence and entrepreneurial resilience. Individual success is inseparable from the basic characteristics of self-confidence, resilience, hope and optimism (Luthans and Youssef, 2004). Therefore, we can explore the internal mechanism of entrepreneurial passion and entrepreneurial success based on the perspective of individual psychological capital.

Psychological capital refers to the individual’s mental state or traits, including the confidence to make efforts to succeed in the challenge, the optimism about the positive attribution of success, the perseverance of hope for the goal, and the resilience to persevere in adversity (Luthans et al., 2005). In the field of entrepreneurship, the psychological capital of entrepreneurs refers to the psychological characteristics of self-confidence, optimism, hope and resilience shown by individuals when facing entrepreneurial challenges (Newman et al., 2021). The psychological stress theory points out that psychological capital affects individual behavior and attitude choices (Goldsmith et al., 1998). Individuals with positive psychological capital can not only correctly handle unexpected problems in entrepreneurship, but also respond flexibly to entrepreneurial challenges, ensure the smooth progress of entrepreneurial activities, and achieve entrepreneurial success (Syed et al., 2020). Therefore, in the process of entrepreneurial success, individual psychological capital plays an important antecedent role.

In conclusion, there must be a connection between entrepreneurial passion, psychological capital and entrepreneurial success, and the relationship between them will be further affected by the support of external entrepreneurial policies. However, our study on entrepreneurial passion and entrepreneurial success from the perspective of psychological capital is rare (Hatak et al., 2021). Therefore, based on resource conservation theory and social processing theory, this article explores the relationship between entrepreneurial passion, psychological capital, entrepreneurial policy support, and entrepreneurial success, reveals the internal mechanism of entrepreneurial passion affecting entrepreneurial success, explores the mediating role of psychological capital, and tests the boundary effect of entrepreneurial policy support, thus complementing and perfecting the related research on entrepreneurial success.

## Theoretical Background and Hypothesis Development

Entrepreneurial passion is the obtainable, conscious, and strong positive emotions that individuals exhibit when they perceive that their entrepreneurial activities are consistent with their own entrepreneurial identity during the process of participating in entrepreneurship (Cardon et al., 2005, 2009). At present, academic circles generally believe that entrepreneurial passion should be divided into two dimensions: one is that entrepreneurs must have positive emotions about entrepreneurial activities; the other is that entrepreneurs must subjectively identify with their entrepreneurial identity (Vignoles et al., 2006). Specifically, entrepreneurial passion is not a general emotion, but needs to rely on activities that have the meaning of entrepreneur identity to stimulate. When individuals perceive their entrepreneurial identity from certain activities, they can experience entrepreneurial passion (Cardon et al., 2013). In addition, Smith et al. (2001) believes that entrepreneurial passion is an inherent trait of entrepreneurs based on the perspective of individual traits; Bird (1989) believes that entrepreneurial passion is a motivation to stimulate entrepreneurs’ behavior based on the perspective of motivation. Different perspectives have different definitions of the connotation of entrepreneurial passion. We chose to use the original Cardon et al. (2005)’s definition as the starting point for this research.

Entrepreneurship success is mainly divided into two perspectives: entrepreneurial success and entrepreneur’s career success (Zhang et al., 2019). Rahman et al. (2015) and Staniewski (2016) believed that entrepreneurial success is the continuous growth of corporate performance and non-performance or reaching a high level in the industry; Lafuente et al. (2013) thought that the success of entrepreneurship mainly depends on the level of marketing, internationalization, financing and sustainable development; Wickham (2006) divided entrepreneurial success into three levels: economic return, psychological success, and social influence. Different types of entrepreneurs pay different attention to the above three levels because of their different entrepreneurial motives. Other scholars believe that the criteria for judging entrepreneurial success should be based on entrepreneurs as a research perspective, and divided into subjective and objective success. The subjective aspect of success is the satisfaction of the company, the high quality of life, the realization of personal value, etc., (Lau, 2002); the objective aspect of success is the factors such as personal income, personal wealth creation, sustainable development of the company, and employee growth rate (Perren, 1999; Amit et al., 2001). It can be seen from the above research that most scholars’s criteria for success are measured from the explicit level (such as the rate of return, financial indicators) and the recessive level (such as influence, etc., non-financial indicators). Explicit financial indicators are divorced from the research object, while implicit indicators are different due to the different characteristics of entrepreneurs. We are based on the research of entrepreneurial success at the individual level; therefore, we choose the explicit indicators from the entrepreneur’s perspective of success for measurement.

Psychological capital reflects the positive mental state of an individual, and contains four dimensions: self-efficacy (believing that one can complete challenging tasks), hope (the ability to persevere toward goals), and optimism (full of positiveness for the future expectations) and resilience (the ability to quickly recover from setbacks and failures) (Luthans et al., 2010). Different from other positive core constructs, psychological capital is regarded as a kind of psychological resource with similar status and exploitability. Existing research also supports this conclusion. For example, Luthans et al. (2010) believed that psychological capital is clearly different from core self-evaluation, positive affect, and big five personality, which is different from relatively fixed personality traits and different from frequently changing personality traits. It is somewhere in between and can be cultivated and developed. Scholars have carried out a lot of research around the four dimensions of psychological capital, and have achieved certain research results. Existing research believes that it is necessary for future research to integrate the four psychological resources into a core construct (i.e., psychological capital) to reflect the basic synergy of resources (Luthans et al., 2005). In addition, psychological capital as a whole construct reflects the positive mental state of the individual, and has a positive impact on entrepreneurs’ entrepreneurial attitude, entrepreneurial behavior, and entrepreneurial performance.

Entrepreneurship policy support refers to the supportive policies provided by administrative agencies (such as government departments) in order to reduce the adverse effects of imperfect systems on entrepreneurial activities. It is an important institutional environment faced by entrepreneurs. Huang et al. (2019) believed that support policies for entrepreneurial activities can be divided into direct support and indirect support. Specifically, direct support includes direct resource support such as financial support, tax incentives, and training; indirect support policy is an environmental policy tool, which mainly provides entrepreneurs with higher government efficiency or service level, such as providing a unified office place, factory building, market and technology information exchange platform, etc., which can create an entrepreneurial support atmosphere for technology entrepreneurs. This study can also discuss the boundary effect of entrepreneurial policy support on entrepreneurial success from these two aspects.

### Entrepreneurship Passion and Entrepreneurial Success

Entrepreneurship passion originates from philosophy and is an individual’s strong tendency toward favorite activities (Vallerand et al., 2003). Baum and Locke (2004) defined entrepreneurial passion as an individual’s love for entrepreneurial activities based on entrepreneurial background. Cardon and Kirk (2015) believed that entrepreneurial passion is the conscious, strong, and positive emotions experienced by entrepreneurs when they participate in entrepreneurial activities. These activities are related to the entrepreneur’s self-identification and prominent role. With the deepening of related research, the importance of entrepreneurial passion for entrepreneurial results has gradually attracted the attention of the academic community.

In the field of entrepreneurial research, entrepreneurial passion positively affects the entrepreneurial process and entrepreneurial success (Bird, 1989; Cardon et al., 2005). The two important dimensions of entrepreneurial passion, the positive emotions and identity of entrepreneurs, are inseparable from entrepreneurial success. First of all, entrepreneurs with positive emotions can continue to strive to achieve success (Samuelsson, 2009), and even this positive state can prompt entrepreneurs to complete their goals and tasks and achieve success in the business, and continue to invest in the next longer-term plan (Heiby, 1995); secondly, entrepreneurs with a sense of identity are more likely to have psychological ownership of the company they create, thereby increasing their sense of responsibility to the company and being able to fight for the success of the company more specifically.

Successful entrepreneurs often emphasize the power of passion. The path to entrepreneurship is full of difficulties. Passion is the driving force that drives entrepreneurs to continue pursuing their goals when encountering difficulties (Cardon and Kirk, 2015). Existing studies have found that individuals with entrepreneurial passion are more aware of the entrepreneurial activities they are engaged in, increase their attention to entrepreneurial goals, and improve their knowledge and skills through hard work. The improvement of ability is helpful to deal with all kinds of difficulties and challenges in the process of starting a business and realize the success of starting a business (Ma et al., 2017). This means that passionate entrepreneurs are more likely to achieve entrepreneurial success in the process of starting a business. Vallerand et al. (2010) found that passionate entrepreneurs have a stronger tendency toward goals and are willing to make great efforts to overcome obstacles to create and develop their own new enterprises, thereby improving corporate performance. Mueller et al. (2017) proposed that entrepreneurial passion can enable entrepreneurs to persevere in the face of difficulties and challenges, and further achieve entrepreneurial success. To sum up, entrepreneurial passion enables entrepreneurs to have a stronger sense of identity with the entrepreneurial activities they are engaged in, to be more determined in their entrepreneurial goals, and to be willing to invest more time and materials to start a business, to cope with the difficulties and challenges in the process of starting a business, and to achieve entrepreneurial success. Based on this, we propose the following hypotheses:

*Hypothesis 1: Entrepreneurship passion has a positive impact on entrepreneurial success.*

### The Mediating Role of Psychological Capital

Psychological capital is an individual’s positive psychological state and characteristics, the source of value creation, the key factor and core asset for obtaining competitive advantage (Zhao et al., 2018), including: when faced with entrepreneurial challenges, have confidence and can make necessary efforts to gain confidence in success; optimism that current and future success can be positively and correctly attributable; perseverance to the goal and the hope of not giving up easily; perseverance in adversity (Aryee et al., 2008). According to the theory of resource conservation, individuals can use their resources to deal with entrepreneurial pressure and meet environmental needs to obtain positive results (Hobfoll, 2002).

In recent years, the empirical discussion on entrepreneurial passion is still in its infancy (Mueller et al., 2017), and there is no in-depth exploration of its impact on psychological capital and entrepreneurial success.

Existing studies have shown that entrepreneurial passion can increase entrepreneurs’ tolerance for risks, and influence entrepreneurs’ cognition through motivational and emotional channels, improve cognitive flexibility, and trigger entrepreneurs to improve their entrepreneurship (Stroe et al., 2018). Based on the theory of resource conservation, individuals tend to obtain resources from outside for individual improvement (Ford, 2007). Entrepreneurship passion, as the driving force of individual entrepreneurship, can further enhance the motivation of entrepreneurs to obtain resources from the outside. The accumulation of resources is conducive to the promotion of entrepreneurial activities. Therefore, it is conducive to enhancing the psychological capital of entrepreneurs.

Existing studies have shown that psychological capital has a significant positive impact on the outcome variables. The higher the level of individual psychological capital, the higher the entrepreneurial performance. Psychological capital and human capital constitute the overall resource capacity required for entrepreneurship. It has been proven that the effect of psychological capital on entrepreneurship is far greater than that of human capital and social capital (Luthans et al., 2010). The overall score dimension of psychological capital has a more significant impact on job performance (Luthans et al., 2005). According to the resource conservation theory, individual resources are scarce and limited, and individual characteristics affect their resource allocation. Therefore, psychological capital, as a positive individual trait, also affects the allocation of resources, that is, psychological capital may affect entrepreneurial activities by affecting individual resource allocation. Research has found that individuals with high psychological capital can make reasonable use of resources to cope with entrepreneurial challenges (Karatepe and Karadas, 2014). Psychological capital’ s entrepreneurial efficacy, familiarity with entrepreneurial procedures, evaluation of entrepreneurial returns, and entrepreneurial preparation have an impact on entrepreneurial success. Entrepreneurs with stronger psychological capital are more challenging and courageous in making decisions, and have greater ability to act (Sequeira et al., 2007), entrepreneurship is more likely to succeed. Based on this, this study believes that psychological capital, as an individual trait, can promote entrepreneurial success by influencing the process of individual resource allocation. Based on this, the following hypotheses are proposed:

*Hypothesis 2: Entrepreneurship passion has a positive impact on psychological capital.*

*Hypothesis 3: Psychological capital mediates the relationship between entrepreneurial passion and entrepreneurial success.*

### The Moderating Role of Entrepreneurial Policy Support

Entrepreneurship passion promotes entrepreneurial success by enhancing individual psychological capital. External policy support has a particularly significant impact on this mechanism. Entrepreneurship policy support is divided into direct support and indirect support. On the one hand, direct policy support. According to resource-based theory, the technological knowledge, skills, and innovation network possessed by entrepreneurs are the key to gaining a competitive advantage, but if other entrepreneurial resources are scarce, the existing resource structure will not be able to adapt to the comprehensive and dynamic requirements of entrepreneurial activities (Zeng et al., 2010). Therefore, policy support can promote the accumulation of individual resources by promoting entrepreneurial passion, which includes both the optimization of the material resources needed for entrepreneurship and the improvement of psychological resources. In addition to general human capital requirements, entrepreneurs must also possess basic entrepreneurial psychological qualities, which are embodied in entrepreneurial resilience, entrepreneurial confidence, and entrepreneurial hope. The government’s entrepreneurial education support increases their entrepreneurial knowledge and at the same time enhances entrepreneurs’ psychological capital.

On the other hand, indirect policy support. First, indirect policy support provides entrepreneurs with a space for mutual learning and cooperation. Specifically, entrepreneurial activities are difficult to rely on technological entrepreneurs to complete independently, and the government-led and constructed regional network of technology parks, high-tech zones, technology alliances, etc., enables technology entrepreneurs in the same area to engage in similar and complementary industries to have more cooperation opportunities (Huang et al., 2019). Close communication and cooperation among technological start-up enterprises is conducive to the flow of explicit information and knowledge resources among enterprises, and promotes the diversity of entrepreneurial resources. At the same time, it brings psychological capital accumulation in entrepreneurial confidence and resilience. At the same time, it is conducive to mutual learning and acquisition of hidden experience and skills among entrepreneurs, strengthening the use of existing resources by entrepreneurs, and enabling them to perceive opportunities more sensitively. Second, the good entrepreneurial atmosphere created by indirect policy support is conducive to entrepreneurs to eliminate negative pressure, improve the level of psychological pressure and the ability to flexibly deal with entrepreneurial challenges, so that they can persist in entrepreneurship until they succeed. The entrepreneurial atmosphere created by the indirect support from the government plays a role in “justifying the name” of entrepreneurs, establishing the entrepreneurial spirit of dare to challenge, compete for the first place, and innovate in the regional environment, and form positive encouragement while enhancing individual psychological capital. Based on this, the following hypotheses are proposed:

*Hypothesis 4: Entrepreneurship policy support promotes the transformation of entrepreneurial passion into individual psychological capital. The higher the level of entrepreneurial policy support, the stronger this promotion relationship.*

### Conditional Process Model

**FIGURE 1:**
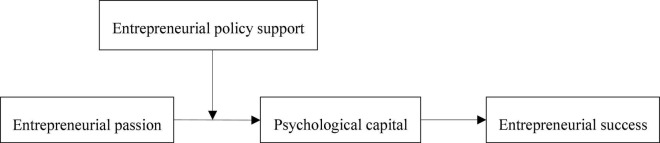
Hypothesized model.

The above theoretical derivation clarifies the role of policy support in moderating entrepreneurial passion and entrepreneurial success, but the principle and mechanism of policy support to enhance the relationship between entrepreneurial passion and entrepreneurial success need to be further explored. Entrepreneurship passion promotes entrepreneurial success by increasing the accumulation of entrepreneurs’ psychological capital. Based on the above assumptions, this research further infers that, compared with low-level entrepreneurial policy support, entrepreneurs with high-level entrepreneurial policy support will generate higher psychological capital when they have entrepreneurial passion, and then increase their entrepreneurial success probability. That is, entrepreneurial policy support plays a moderating role in the mediating effect of psychological capital. This paper constructs a moderated mediation model, as shown in Figure 1. Based on this, the following hypotheses are proposed:

*Hypothesis 5: Entrepreneurship policy support moderates the mediating role of psychological capital between entrepreneurial passion and entrepreneurial success. The higher the level of entrepreneurial policy support, the stronger the mediating relationship.*

## Materials and Methods

### Participants and Procedures

In the questionnaire design of this study, in order to avoid common method bias, a total of 93 innovative and entrepreneurial teams in different fields were selected. The team is mainly distributed in industrial entrepreneurship parks and college entrepreneurial incubation centers in Wuhan, Guangdong, Shanghai and other places. The data was collected at three time points. At T1 (February 2021), for 93 teams, 523 questionnaires were distributed through e-mail, telephone, and door-to-door visits, and the purpose of the questionnaire was explained to ensure that the interviewees anonymity and privacy. Time 1 collected data on entrepreneurial passion and demographic variables. After excluding invalid questionnaires, 512 valid questionnaires were obtained; At T2 (May 2021), 512 questionnaires were tracked and investigated to obtain psychological capital and entrepreneurial policies support questionnaire data. After excluding invalid questionnaires, 483 valid questionnaires were obtained; At T3 (September 2021), 483 questionnaires were tracked to obtain questionnaire data for entrepreneurial success. After invalid questionnaires were removed, valid data was obtained as 455 shares. Among the surveyed samples, women accounted for 41.9% and men accounted for 58.1%; Employes under 20 accounted for 10.2%, 21–30 years old accounted for 39.5%, 31–40 years old accounted for 33.4%, and 40 years old and above accounted for 16.9%; The proportion of associates and below was 25%, the proportion of bachelor’s degree was 39.4%, the proportion of master’s degree and above was 35.6%.

### Measures

In order to ensure the reliability and validity of the measurements, this study adopted the mature scale widely used in authoritative journals at home and abroad to measure related variables. According to the cross-cultural translation-back translation procedure, through expert discussion and modification and optimization, we strove to achieve accurate semantic expression, standard expression form, and conform to Chinese language standards, and finally finalized the formal questionnaire. Unless otherwise specified, all scale items were measured using the Likert 5-point scale method. 1 represents “*strongly disagree*” and 5 represents “*strongly agree*.”

#### Entrepreneurship Passion

The scale developed and validated by Cardon et al. (2005) was adopted in the study, it included 13 items, such as “It is very exciting to have your own business” and “It is very exciting to discover new opportunities and commercialize them.” In this study, the Cronbach’ s α was 0.853.

#### Psychological Capital

The four-dimensional 19-degree item scale developed by Luthans et al. (2005) was used to measure individual self-confidence, optimism, hope and resilience. Sample items were: “I can think of many ways to achieve my goals” and “I am optimistic about what will happen in the future.” In this study, the Cronbach’ s α was 0.880.

#### Entrepreneurship Policy Support

We measured entrepreneurship policy support with a 5-item scale from Li and Atuahene-Gima (2001), see also Ying et al. (2018). Sample items: “The local government has formulated and implemented policies that are beneficial to business operations such as factory buildings, office space, tax reductions and exemptions,” “The local government has provided the required technical information and other technical support.” In this study, the Cronbach’ s α was 0.842.

#### Entrepreneurship Success

To assess entrepreneurship success, we used the scale developed by Rahman et al. (2015), it included 9 items, such as “Our company’s sales continue to grow” and “Customers trust our company’s products and services.” In this study, the Cronbach’ s α was 0.812.

#### Control Variables

This research refers to the research of Zhang et al. (2019). We used “gender” (Male = 1; Female = 0), “age” (“Under the age of 18” = 1; “18–30” = 2; “30–40” = 3; “40–50” = 4; “50 years of age or older” = 5)and “education” (“Under the high school” = 1; “high school” = 2; “Bachelor’s degree” = 3; “Graduate degree” = 4) as control variables to reduce their impact on entrepreneurial passion and entrepreneurial success.

## Results

### Confirmatory Factor Analysis

A confirmatory factor analysis was carried out on the scale using AMOS 24.0. As shown in Table 1, the fitting of the four-factor model is optimal: *x*^2^/*df* is 1.256, TLI and CFI are greater than 0.9, and NFI and GFI are greater than 0.8. It shows that the scale has a relatively ideal external structure validity, and the follow-up analysis can be continued.

**TABLE 1:** The result of confirmatory factor analyses.

Model	*χ^2^/df*	RESEA	GFI	TLI	CFI
Four-factor model^a^	1.256	0.030	0.900	0.981	0.983
Three-factor model^b^	4.504	0.110	0.836	0.739	0.758
Two-factor model^c^	5.414	0.123	0.701	0.672	0.694
One-factor model^d^	7.211	0.146	0.637	0.538	0.569

*N = 455. χ^2^ = chi-square statistic; CFI, comparative fit index, TLI, Tucker–Lewis index, RMSEA, root mean square error of approximation, and GFI, goodness-of-fit index.*

*^a^entrepreneurship passion, psychological capital, entrepreneurial policy support, entrepreneurial success.*

*^b^entrepreneurship passion + psychological capital; entrepreneurial policy support; entrepreneurial success.*

*^c^entrepreneurship passion + psychological capital + entrepreneurial policy support; entrepreneurial success.*

*^d^entrepreneurship passion + psychological capital + entrepreneurial policy support; entrepreneurial success.*

### Data Aggregation Test

Entrepreneurship policy support questionnaires were answered by individuals, so before aggregated to the policy level, the consistency of the variables within the group needs to be tested. Using R_*wg*_ index and ICC index test, the values of ICC (1) and ICC (2) supported by entrepreneurial policies are 0.195 and 0.552, respectively, which were in line with James et al. (1993) that ICC(1) should be higher than the 0.12 standard. Schneider et al. (1998) identified ICC (2) higher than 0.47 standard. The average value of R_*wg*_ was 0.84, which was greater than the standard of 0.7, indicating that it was feasible to aggregate low-level data to higher levels.

### Descriptive Statistics

We used Pearson correlation coefficient for correlation analysis, as shown in Table 2. Entrepreneurship passion was positively correlated with psychological capital (*r* = 0.631, *p* < 0.001), and positively correlated with entrepreneurial success (*r* = 0.324, *p* < 0.001); psychological capital was positively correlated with entrepreneurial success (*r* = 0.729, *p* < 0.001).

**TABLE 2:** Means, standard deviations, and correlations among the variables.

	*M*	*SD*	1	2	3	4	5	6	7
*level 1*									
Gender	1.341	0.473	1						
Age	2.662	0.978	0.010	1					
Education	2.601	1.003	0.061	–0.003	1				
Entrepreneurial passion	2.893	0.387	0.039	–0.014	0.113	1			
Psychological capital	4.107	0.714	0.073	0.082	0.156	0.213***	1		
Entrepreneurial success	4.461	0.666	0.037	0.067	0.074	0.133***	0.153***	1	
*level 2*									
Entrepreneurship policy support	3.762	0.672	0.065	0.036	0.064	0.034	0.444***	0.146*	1

*p < 0.05, **p < 0.01, and ***p < 0.001.

### Mediating Effect of Psychological Capital

Using HLM 7.0, we analyzed the mediating role of psychological capital with entrepreneurial passion and entrepreneurial success. The regression analysis results of entrepreneurial passion, psychological capital, and entrepreneurial success were shown in Table 3. Entrepreneurship passion was positively correlated with psychological capital (*r* = 0.220, *p* < 0.001), and positively correlated with entrepreneurial success (*r* = 0.200, *p* < 0.001). Therefore, Hypothesis 1 and Hypothesis 2 were supported.

**TABLE 3:** Analysis results of main effects and mediation effects.

Variable	Psychological capital		Entrepreneurial success		
		
	M1	*M*2	*M*3	*M*4	*M*5
Gender	0.052	0.072	0.060	0.052	0.720
Age	0.033	0.042	–0.039	0.330	0.460
Education	0.042	0.035	0.041	0.420	0.075
Entrepreneurial passion		0.220***		0.200***	0.160
Psychological capital					0.210***
R^2^	0.030	0.270	0.191	0.030	0.264
ΔR^2^		0.015***	0.161***		0.234***

*p < 0.05; **p < 0.01; and ***p < 0.001.

As shown in Table 3, psychological capital was positively correlated with entrepreneurial success (*r* = 0.210, *p* < 0.001), which preliminarily verified the mediation effect. Furthermore, we took the Monte Carlo method and sample 5,000 times to test the mediation effect. The 95% confidence interval was [0.096, 0.201], excluding 0. Therefore, Hypothesis 3 was supported.

### Moderating Effects of Entrepreneurship Policy Support

**FIGURE 2:**
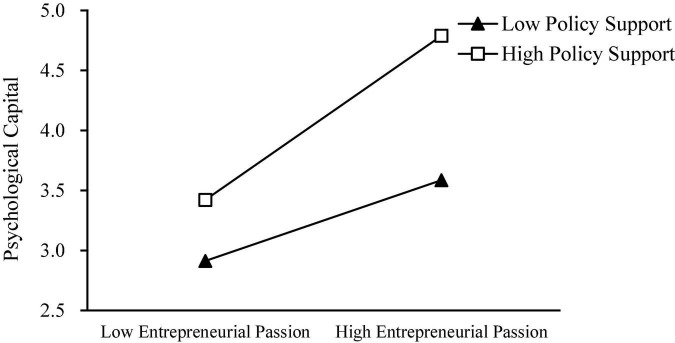
Moderating effect of policy support between entrepreneurial passion and psychological capital.

Entrepreneurship policy support is a level 2 variable, while entrepreneurial passion, psychological capital and entrepreneurial success are level 1 variables. Therefore, we used HLM 7.0 to analyze the data, and the results were shown in Table 4. We set up a null model with psychological capital, and constructed an interactive item of entrepreneurial passion and entrepreneurial policy support. As shown in Table 4, the interaction term was positively significant for psychological capital (β = 0.212, *p* < 0.01). In order to further demonstrate the moderating effect of entrepreneurial policy support, we drew a diagram of the moderating effect, as shown in Figure 2. Under the high level of entrepreneurial policy support, the impact of entrepreneurial passion on psychological capital was stronger than that of the low-level entrepreneurial policy support. Therefore, Hypothesis 4 was supported.

**TABLE 4:** Cross-level regression analysis results.

Variable	Psychological capital		
	
	M6(Null)	M7	M8
Intercept	3.131***	3.122***	3.131***
Entrepreneurial passion		0.161**	0.180**
Entrepreneurship passion*Entrepreneurship policy support			0.212**
R^2^_*with*_		0.161	0.103
ΔR^2^_*between*_		0.254	0.463***

*p < 0.05; **p < 0.01; and ***p < 0.001.

### Moderated Mediation Effects

We calculated the indirect effect of entrepreneurial passion on entrepreneurial success through psychological capital under different conditions (plus or minus one standard deviation) of entrepreneurial policy support, and obtained a 95% confidence interval. As shown in Table 5, the confidence interval did not contain 0, indicating that the conditional process model was established. Therefore, Hypothesis 5 was supported.

**TABLE 5:** Test results of conditional process model.

Moderator	LEVEL	Effect	Standard error	95% CI
Entrepreneurship policy support	HIGH	0.110	0.032	[0.052, 0.141]
	LOW	0.160	0.043	[0.190, 0.263]

*Bootstrap size = 5,000.*

## Discussion

### Theoretical Contributions

First, this research explores the path of entrepreneurial passion on entrepreneurial success, and provides a systematic theoretical reference for follow-up research. Due to the controversy over the definition of entrepreneurial success and the measurement of scales in academic circles, the research on entrepreneurial passion and entrepreneurial psychological capital has just started, and the number of related research documents is relatively small. Some aspects of the two variables of entrepreneurial passion and psychological capital are more personality traits. The research on is biased toward psychology, which aggravates research difficulties (Karatepe and Karadas, 2014). This research provides an important reference for the empirical research on entrepreneurial success, psychological capital, and entrepreneurial passion.

Second, entrepreneurship is an internal social process, and its success depends to a large extent on the support of social policies. Existing studies on policy support for entrepreneurial success mostly use explicit indicators (Holland and Shepherd, 2013). Although policy support can generate a driving force for entrepreneurs, not all entrepreneurs feel achievement and satisfaction (Smes et al., 1995). This study takes entrepreneurs as the research object, examines the boundary effect of policy support on the relationship between entrepreneurial passion, psychological capital, and entrepreneurial success, enriches the research perspective of policy support. While supporting the conclusion of Holland and Shepherd (2013)’s study, it also confirms policy support promotes the positive effect of entrepreneurial passion on psychological capital.

Thirdly, this research is based on the theory of resource conservation and examines the positive effects of entrepreneurial passion through psychological capital on entrepreneurial success from the perspective of individual psychological capital. Applying the theory of resource conservation from the fields of management and psychology to the field of entrepreneurship is an attempt to merge the fields, and it is also an extension and test of the theory of resource conservation.

### Practical Implications

The First, this research proves the core role of entrepreneurial passion in influencing entrepreneurial behavior and entrepreneurial success. If passion can really predict entrepreneurial success, educators, tutors and other entrepreneurs should work harder to help new entrepreneurs cultivate entrepreneurial passion and encourage entrepreneurs to identify and possess passion. The government can strengthen entrepreneurial propaganda, introduce policies to encourage entrepreneurship, create a good environment for entrepreneurship, and in turn stimulate the people’s entrepreneurial passion.

Second, psychological capital also has a crucial impact on entrepreneurial success. Entrepreneurs should realize the importance of entrepreneurial passion and persistence, keep their passion and passion for entrepreneurial activities, and constantly obtain the material and psychological resources needed for entrepreneurial activities from the external environment, so as to ensure the smooth progress of entrepreneurial acquisition.

Third, entrepreneurial policy support can promote the relationship between entrepreneurial passion and psychological capital. Entrepreneurship passion is the core driving force of entrepreneurs’ entrepreneurial behavior, which is not only affected by the inherent attributes of entrepreneurs, but also by the policy environment. Therefore, on the one hand, the government can provide positive feedback to entrepreneurs through commendations, rewards and other forms to stimulate entrepreneurs’ positive emotions; on the other hand, the government can set an example of typical successful entrepreneurs, and strengthen publicity through effective channels, so as to improve the entrepreneurs’ sense of identity as self-entrepreneurs and enhance their psychological capital. From an entrepreneur’ s point of view, “Good wind, with its strength, sends me to Qingyun.” While improving their own psychological capital, entrepreneurs should also actively pay attention to relevant support policies and learn from relevant policies and regulations on the use of policies to promote entrepreneurship success.

### Limitations and Future Directions

Although our research has done a lot of work, there are still limitations and deficiencies that cannot be avoided.

First, data collection. Although the questionnaire in this research adopted an anonymous method for data collection, in view of the existence of the social approval effect, entrepreneurs would ideally exaggerate the success of the business, which would affect the objective and authenticity of the data. Therefore, future research can adopt methods such as experiments and financial data for data collection to improve the objectivity of variable measurement.

Second, this article takes psychological capital as a single mediating variable and only considers the positive effects of individual internal factors on entrepreneurial success. Since entrepreneurial success is the result of multiple factors, relying only on a single internal factor may not fully explain its internal mechanism. Therefore, we call for future research to take into account factors at the individual level, organizational level, and social level in order to interpret the impact mechanism of entrepreneurial passion on entrepreneurial success in a more comprehensive manner.

Third, this study used the environmental factor of entrepreneurial policy support as a moderating variable to explore its role in moderating the relationship between entrepreneurial passion and entrepreneurial success. The improvement of psychological capital is also affected by non-environmental factors such as individual characteristics and entrepreneurial events. Therefore, in the next stage of research, more relevant moderating factors can be included to enrich the research content and make the research more scientific and rigorous.

## Conclusion

Based on literature review and empirical research tests, we constructed a conditional process model and reached the following research conclusions:

First, the test of direct and indirect effects. First of all, entrepreneurial passion has a significant positive impact on entrepreneurial success. It shows that the strong emotions of passion can directly affect the success of entrepreneurs. Secondly, entrepreneurial passion has a significant positive impact on individual psychological capital, and psychological capital has a significant positive impact on entrepreneurial success. It shows that entrepreneurial passion can bring about the accumulation of entrepreneurs’ psychological capital and enhance the precious qualities of entrepreneurial confidence and entrepreneurial resilience, which need to be paid attention to by entrepreneurs. Finally, psychological capital partially mediates the relationship between entrepreneurial passion and entrepreneurial success. It shows that not all individuals with entrepreneurial passion will succeed in entrepreneurship. Many entrepreneurs need to transform entrepreneurial passion into psychological capital of confidence, hope, optimism and resilience to support their persistence.

Second, the moderating effect test. Entrepreneurship policy supports positive moderating of the positive relationship between entrepreneurial passion and psychological capital. Policy support not only provides entrepreneurs with certain entrepreneurial resources, but can also increase entrepreneurs’ self-confidence and other psychological capital, which helps entrepreneurs achieve success in entrepreneurial activities. Although we have not put forward the hypothesis that entrepreneurial support is between entrepreneurial passion and entrepreneurial success, our data support this view. Entrepreneurship policy support is undoubtedly a huge help to entrepreneurs, but whether it can directly promote entrepreneurship success still requires individual effort and persistence.

Third, conditional process model testing. Entrepreneurial policy support positive moderating of entrepreneurial passion through psychological capital’s mediating role in entrepreneurial success. Entrepreneurship, as an individual trait, needs to be transformed into psychological capital suitable for entrepreneurship with the support of the outside world. At the same time, when the internal transformation mechanism from entrepreneurial passion to entrepreneurial process is supported by entrepreneurial policies, entrepreneurs are more likely to succeed.

## Data Availability Statement

The original contributions presented in the study are included in the article/supplementary material, further inquiries can be directed to the corresponding author.

## Ethics Statement

Ethical review and approval was not required for the study on human participants in accordance with the local legislation and institutional requirements. The authors declare that they strictly adhered to the APA guidelines on ethical research practices. Written informed consent for participation was not required for this study in accordance with the national legislation and the institutional requirements. The patients/participants provided their online informed consent to participate in this study, which stated the voluntary nature of participation, and assurance of confidentiality and anonymity.

## Author Contributions

All authors listed have made a substantial, direct, and intellectual contribution to the work, and approved it for publication.

## Conflict of Interest

The authors declare that the research was conducted in the absence of any commercial or financial relationships that could be construed as a potential conflict of interest.

## Publisher’s Note

All claims expressed in this article are solely those of the authors and do not necessarily represent those of their affiliated organizations, or those of the publisher, the editors and the reviewers. Any product that may be evaluated in this article, or claim that may be made by its manufacturer, is not guaranteed or endorsed by the publisher.
